# Tissue Liquefaction Liposuction for Body Contouring and Autologous Fat Transfer: A Retrospective Review Over 3 Years

**Published:** 2016-12-24

**Authors:** Zachary M. Borab, Christopher P. Godek

**Affiliations:** ^a^Wyss Department of Plastic and Reconstructive Surgery, New York University School of Medicine, New York; ^b^Personal Enhancement Center, Toms River, NJ

**Keywords:** liposuction, lipoplasty, autologous fat grafting, adipose fat transfer, body contouring

## Abstract

**Objective:** Tissue liquefaction lipoplasty is a novel, low-energy method cleared for use in aesthetic body contouring and autologous fat transfer. This is a retrospective review of the clinical effectiveness and safety of a liquefaction lipoplasty system for liposuction and autologous fat transfer. **Methods:** A retrospective review was done evaluating all liquefaction lipoplasty procedures with or without autologous fat transfer performed by a single surgeon (March 2013 to June 2016). Patient demographics, operative details, and any complications were tabulated from patient charts. A typical case reported is presented with pre-/postoperative photographs. **Results:** Two hundred fifty-five consecutive liquefaction lipoplasty procedures were performed over 39 months. The average lipoaspirate volume was 1208 ± 991 mL and the average fat graft volume was 322 ± 277 mL. The overall complication rate was 9 of 255 (3.52%). There were 2 episodes of seroma (0.78%) that were aspirated and 2 episodes of cellulitis (0.78%) that responded to oral antibiotics. In the autologous fat transfer cohort, there were 5 of 103 (4.85%) cases of mild to moderate fat necrosis, with 1 patient requiring return to the operating room for removal of an oil cyst. No revisions of donor sites were required. **Conclusions:** Liquefaction lipoplasty appears safe for liposuction and autologous fat transfer, with a complication profile that is comparable with other widely used forms of suction-assisted liposuction. The liquefaction lipoplasty technology also provides potential time savings in the operating room that can minimize surgeon fatigue when harvesting large volumes of high-quality fat. Liquefaction lipoplasty appears to have advantages for both the patient and the surgeon, and further studies are underway.

In recent years, the popularity of liposuction and autologous fat transfer (AFT) procedures has reached an all-time high. Liposuction is the second most commonly performed cosmetic procedure and has increased 5% from 2014 to 2015. AFT is quickly being added to both cosmetic and reconstructive surgeons’ practices, where an estimated 62% of the American Society of Plastic Surgeons members use AFT at least part of the time.^1^ As indications for AFT expansion to areas outside of contour deformity correction, this trend will likely continue.^2-4^ Today, much variety exists in fat harvesting, processing, and injection techniques.^5^ With so many options to choose from and very little comparative research in the literature, this has left us in a current state of uncertainty as to which method is optimal for AFT.

All existing modalities of suction-assisted lipoplasty (SAL) cut and shear tissue at some point during adipose extraction, leaving the patient vulnerable to development of pain, ecchymosis, seroma, and hematoma.^6^ For the surgeon, SAL is labor-intensive and requires manual production of mechanical energy to shear the adipose tissue into smaller clusters for suction aspiration. Despite recent waves of advances in SAL technology, some modalities remain difficult to use and present poorer safety profiles when compared with SAL. Ultrasound energy sources, for example, can result in scarring, fibrosis, burns, as well as skin irregularities, which may be difficult to repair. Ultrasonic energy can also result in long-term pain and overall dissatisfaction with the cosmetic result. Complications such as divoting may result from overresection of fat, whereas visible lumps may be seen with underresection of fat.^7^ Some of the most devastating complications can be seen with higher energy sources such as laser-assisted lipoplasty, which often require a longer operative time, can be less precise, and can also result in cutaneous burns.^8,9^

In 2012, a device utilizing a novel energy source called Tissue Liquefaction Technology (TLT) (HydraSolve, Andrew Technologies, Tustin, Calif) was cleared by the US Department of Food and Drug Administration (FDA) to be used for liquefaction and aspiration of localized subcutaneous fatty deposits for the purpose of aesthetic body contouring.^10^ This new category of lipoplasty procedure—tissue liquefaction liposuction (TLL)—utilizes a stream of warmed (37-55°C), low-pressurized (300-1100 psi), and pulsed saline solution to liquefy only targeted adipose tissue while other structures are preserved including the skin, blood vessels, nerves, and connective tissue. In April 2013, TLL also received FDA 510 (k) clearance for autologous fat transfer (AFT) so that the adipose tissue harvested can be reinjected into the same patient for reconstruction, rejuvenation, or augmentation. No other liposuction device has been approved for this use to date.

A distinctive feature of TLL is the synergistic action of heat and pressure exerted by the sterile saline. While the thermal energy alone is not enough to burn tissue and the mechanical energy alone is not enough to cut tissue, the combined energy is sufficient to liquefy fat. This novel technology has been in use to liquefy and remove cataracts in a safe and effective manner since 2003.^11-17^ TLL is a low-energy system that achieves target tissue specificity, allowing liposuction to be performed in a new manner that may be gentler, less traumatic, and more precise. In addition, fat harvested with this device contains a high percentage of viable cells with low levels of contaminants^18^ and can be rapidly produced in the large quantities needed for large-volume AFT.

This study is a retrospective review to report on the clinical effectiveness and safety of an integrated TLL system (HydraSolve) for liposuction with or without AFT over 3 years.

## METHODS

### Patients

This retrospective study represents a compilation of results of liposuction with or without AFT performed by a single surgeon (C.G.) from March 2013 to June 2016 using TLL. From 255 consecutive procedures, data were grouped into liposuction alone and liposuction with AFT. Patient demographics and operative details including sex, age, TLL setting, tumescent volume, lipoaspirate site, lipoaspirate volume per site, total lipoaspirate, AFT site, AFT volume per site, and any complications (seroma, fat necrosis, hematoma, cellulitis, contour irregularities, venous thromboembolism, revision surgery, and hospital admission) were tabulated from patient records. A Microsoft Excel data extraction sheet was used for data collection and descriptive statistics (Microsoft Corporation, Redmond, Wa). Preoperative and postoperative photographs were selected at various time points to demonstrate long-term clinical results. In addition, a breast reconstruction case report using TLL for AFT with operative details is presented.

### Surgical technique using TLT

**Figure 1:**
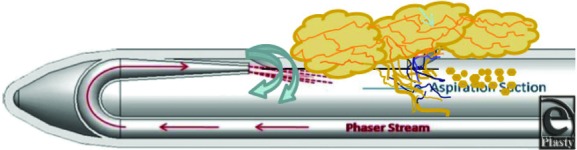
Schematic diagram of the TLL harvesting cannula tip and how the technology combines 3 forces (heat, water pressure, and suction) to selectively remove fat cells.

Liposuction was performed with a TLL system (HydraSolve, Andrew Technologies). Briefly, fat is typically harvested through a “super wet” tumescent technique using a 3- or 4-mm cannula and at a low-power setting. Small incision sites (2-3 mm) were used without the need for skin protection ports. The integrated TLL system was used on 1 of 3 power settings depending on the site (Table 1). The cannula aperture edge is manufactured with a rounded radius of curvature so that it is unable to cut tissue (Fig 1). A variety of reusable, sterilizable cannula designs with single or multiple apertures increase versatility and allow effective treatment of various areas. We used the recommended stroke rate (1 stroke every 2 seconds) for optimal fat harvesting. The fat was collected in a sterile canister and the excess water decanted. Any excess fluid was removed with either a handheld or electric centrifuge at 3000 rpm for 2 minutes and then loaded into 60 mL syringes. A 2-mm single, open-ended cannula was used for injection following needle-band release of scar tissue with a 16-gauge needle for breast reconstruction cases. For AFT to the face, we used the high setting to create microscopic fat for injection. A 1 mL syringe with a 20- or 22-gauge needle was used to transfer fat into various facial sites. The device recorded both the tumescent infused volume and the aspiration volume.

## RESULTS

### Overall

Over a 3-year period, 255 consecutive TLL procedures were conducted in 218 unique patients (203 female, 15 male) by a single surgeon (C.G.) involving 18 anatomical sites. The average age was 48 ± 12 years old (range, 18-74 years). The average lipoaspirate volume was 1208 ± 991 mL (range, 100-4300 mL) (Table 2). AFT was performed in 103 of these procedures. There were no hospital admissions, and the overall adverse event profile was limited to 2 episodes of seroma (2/255; 0.78%), 2 episodes of cellulitis (2/255; 0.78%), and 5 episodes of fat necrosis (5/103; 4.85%) (Table 3).

### Liposuction alone

All patients received “super wet” tumescent infiltration using approximately 1 mL of tumescent fluid per 1 mL of aspirate. The tumescent volume ranged from 100 mL to 5 L. The most common liposuction sites were the flanks (n = 156) and abdomen (n = 114) (Table 2). The average lipoaspirate volume for each type of procedure varied from 30 mL (neck) to 1029 mL (buttocks). The minimum and maximum lipoaspirate volumes were 15 mL (submental) and 2500 mL (anterior thighs), respectively. The maximum lipoaspirate volume in a single surgery was 4300 mL during liposuction of a female patient involving the anterior thighs (2500 mL), knees (1200 mL), anterior tibia (300 mL), and calves (300 mL).

**Figure 2:**
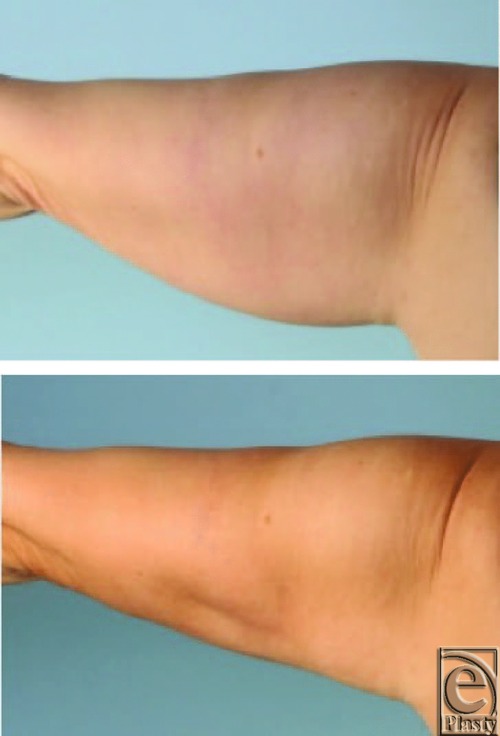
TLL results using “high” setting. (Top) A preoperative photograph demonstrating loose upper arm skin accompanied with excess fat, and (bottom) a postoperative photograph demonstrating an acceptable cosmetic result with good skin retraction.

In general, liposuction without AFT was usually performed on a medium setting; those to be followed by AFT were most often performed on a low setting. The high setting used for 13 procedures without AFT resulted in good to excellent skin retraction in a variety of procedures, including liposuction of the arms (Fig 2), abdomen, and submental area.

All procedures involved liposuction, some of which involved mega volumes of lipoaspirate. When analyzing liposuction site morbidity, only 2 patients experienced postoperative adverse events. Two patients developed seroma (2/255; 0.78%), each about 15 mL by volume, and both resolved with 2 rounds of needle aspiration.

### Autologous fat transfer

Of the 255 TLL procedures, 103 involved subsequent AFT to various recipient sites. The most common location for AFT was to the breast/chest wall (n = 63), followed by face (n = 25), and other body sites (n = 17). Twelve patients had more than 1 AFT recipient location: 2 sites (n = 9), 3 sites (n = 1), and 4 sites (n = 2). Twelve patients underwent more than 1 stage of AFT (range, 2-5 stages). The average volume of fat transferred was 322 ± 277 mL (range, 1-1420 mL). Average AFT volumes based on anatomical areas were as follows: facial, 11 mL; body, 481 mL; and breast, 376 mL.

Large-volume AFT (>100 mL) was performed in 72 patients. The low setting used for the majority of procedures with AFT resulted in good take and contour in a variety of procedures, including AFT to irradiated sites.

Complications were only seen in patients undergoing breast surgery. Five patients developed mild to moderate palpable fat necrosis (5/103; 4.85%), and 1 patient required return to the operating room for removal of an oil cyst (1/103; 0.97%). No revisions of donor sites were required.

### Case report: Utilization of TLL for AFT in breast reconstruction

**Figure 3:**
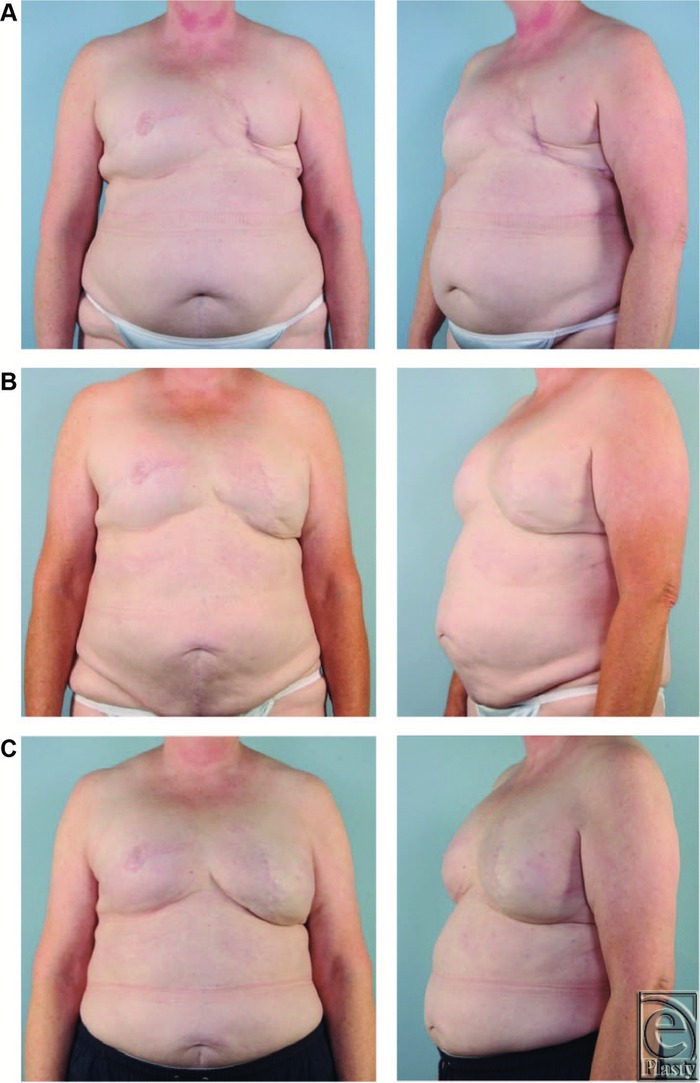
Photograph series using TLL for AFT breast reconstruction. (Top) Images showing failed left-sided implant reconstruction after implant removal and before Brava tissue expansion. (Middle) Images showing 1-year follow-up photographs after 5 rounds of fat grafting using TLL. (Bottom) Images showing 2-year follow-up photographs demonstrating successful long-term graft survival.

A 56-year-old woman received a diagnosis of cancer of the right breast 6 years prior. At that time, she underwent right mastectomy with placement of a tissue expander. This was followed by exchange to 650 cc silicone implant for right breast reconstruction and concurrent left breast reduction using inverted-T incision with inferior pole technique to achieve symmetry. Unfortunately, she developed ductal carcinoma in situ with microinvasion in her left breast 4 years later. After left-sided vertical mastectomy, her first tissue expander failed secondary to infection and was removed. After debridement and resolution of the symptoms, a second tissue expander was placed and later exchanged with a 650 cc implant. Within 2 weeks, there was again evidence of infection and the implant was removed. Now, 6 years after being diagnosed with breast cancer, she had a successful right breast reconstruction and a failed left breast reconstruction (Fig 3*a*).

At this point, we had essentially exhausted implant-based reconstruction techniques and offered alternative autologous tissue options for further repair. She elected to pursue several rounds of fat grafting instead of a pedicled flap or free tissue transfer. Next, the left breast was fit with a Brava dome external tissue expander device (Brava, LLC, Miami, Fla). This was worn only in the preoperative period for 6 weeks, 8 hours per day, in an effort to best prepare the recipient site to receive and support large volume of fat grafts. Over a 2-year period, she received 5 rounds of AFT using the TLL system. Fat was harvested from various sites including abdomen, flanks, posterior hips, thighs, saddlebags, bra line, and lateral chest wall. After 4 AFT procedures, 250, 300, 250, and 270 mL were transferred to the left breast, respectively. In the fifth and final round of AFT, 180 mL of fat was grafted to her left breast and 120 mL to her right breast for symmetry. In total, she received 1250 mL of fat to her left breast over 2 years, with 5 procedures to adequately reconstruct the defect. She experienced minimal fat necrosis in the recipient site and no complications in any of the donor sites. Figure 3*b* shows 1 year after her final round of AFT. Now, 2 years after her final round of AFT, the volume of her left breast is equivalent if not slightly greater than her right breast, which was reconstructed with a 650 cc implant (Fig 3*c*).

## DISCUSSION

Overall, we found TLL to be a reliable and safe method for harvesting and grafting adipose tissue. More than 250 cases were performed without incidence of hematoma, deep venous thrombosis, or hospital admission. With only 9 patients experiencing complications after TLL, our rate of 3.52% (9/255) is much less than what has been reported in recent literature (up to 8.6%) using other liposuction methods.^7^

Without the use of imaging modalities, which can falsely increase incidence rates, we detected 5 episodes of fat necrosis on physical examination. Four of these were managed conservatively and 1 required an operation to remove an oil cyst. Using TLL, our rate of fat necrosis (4.85%) is comparable with the lower end of the published fat necrosis rates, which range from 3.9%^19^ to 47%^20^ of patients. Despite our series averaging 327 mL, and could be described as “large-volume AFT” (>100 mL), we were surprised to observe that the risk of fat necrosis did not increase as larger volumes of fat were transferred. This results is in agreement with a study by Dolen et al,^21^ who describe their experience using TLL for AFT in breast reconstruction. Not surprisingly, they experienced a much higher rate of fat necrosis (22.1%) likely because they used magnetic resonance imaging and ultrasound scan for postoperative surveillance.^21^

Other postoperative morbidity was extremely limited. There were 4 other incidences of complications. Two patients developed seroma, both containing roughly 15 mL of serous fluid and both resolved after 2 rounds of needle aspiration. In addition, 2 patients developed cellulitis, which resolved with oral antibiotics.

### Safety and proposed mechanism of action

**Figure 4:**
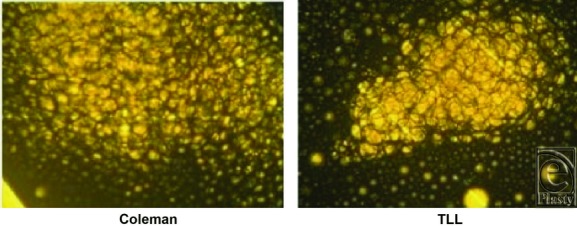
TLL lipoaspirate clump size is reduced compared with Coleman lipoaspirate. Photomicrographs of the supernatant from the lipoaspirate of fat cells harvested by TLL (right) and Coleman technique (left).

**Figure 5:**
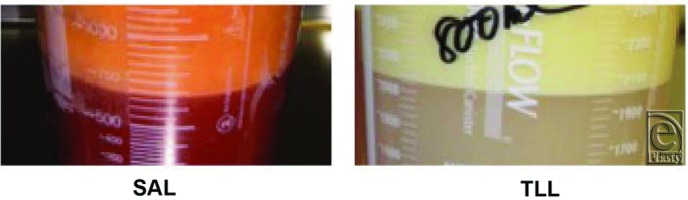
Photographs demonstrating “clean” fat harvested using TLL (right) versus traditional SAL (left).

The excellent safety profile using this device may be related to several factors. The ability to target and harvest fat without forceful thrusting likely plays a role in the reduction of trauma inflicted on the tissue.^12^ At the cellular level, liquefaction of fat tissue by the energized saline stream occurs by partial separation of the cells from one another (disaggregation). Under normal conditions, fat cells attach noncovalently to each other, either directly by adhesive glycoproteins that are located on the surface of fat cells or indirectly via adhesive glycoproteins in the extracellular matrix. The temperatures and water pressure levels used in TLL are key to gently separate some, but not all, of the cells.^22^ This partial disaggregation results in a lipoaspirate that is a multiple-cell suspension containing small clusters consisting of 100 to 400 fat cells in a liquid medium (Fig 4).^23^ The suspension has a soup-like consistency and is relatively devoid of oil, blood, and connective tissue debris. As evidenced by the lipoaspirate contents, the combined forces of pressure, heat, and suction in TLL may serve as an optimal way to harvest fat without damaging the epithelial, intra-abdominal, and neurovascular structures of the donor site that are often responsible for the complications observed with liposuction procedures (Fig 5).

### TLT in other fields

The TLL liposuction device is based on the same patented TLT that has been in use since 2003 for precision cataract surgery. Tens of thousands of treatments around the world have established tissue liquefaction as a method that allows surgeons to remove cataracts with target tissue specificity. The safety and effectiveness of TLT in cataract surgery for 687 patients have been documented in 18 articles published in peer-reviewed ophthalmic journals, including 4 randomized clinical trials^11,13,16,17^ and 1 prospective comparative trial.^14^ No complications were reported in any of those 687 cases when the handpiece tip was inside the eye, actively liquefying and aspirating the cataract.

### Limitations of this study

This is an uncontrolled retrospective chart review of patients treated by TLL with or without AFT, some with long-term follow-up. Because of the nature of the study design, there is potential for bias. The operative data points and postoperative complications were extracted manually from medical records. And although the senior author reported high surgeon and patient satisfaction with long-term cosmetic results, these outcomes were not evaluated using a validated tool. These limitations may play a role in the interpretation of results. In addition, there was no control group to compare outcome measures directly with. Without a control group, it may be difficult to accurately interpret outcome measures from one study to another due to numerous uncontrolled variables such as patient demographics, harvesting sites, harvesting technique, processing technique, and how outcomes were reported. However, the purpose of this study was to introduce a new category of technology to the fields of lipoplasty and AFT and to highlight its safety profile through a retrospective review.

### Future directions

To confirm the observations in the retrospective study presented here, a prospective, randomized, multicenter, blinded trial with a planned enrollment of 30 patients has been initiated. This study is designed as a contralateral study comparing SAL on one side with TLL on the other side. Results will be assessed for statistically significant differences in pain, bruising, swelling, and wound healing.

## CONCLUSION

This is a primary report of a single surgeon's experience using TLL for body contouring and AFT. On the basis of this initial report, TLL appears to have advantages for both the patient and the surgeon. The device has an impressive safety profile and has been shown to be extremely effective, with a novel and efficient mechanism for fat extraction. Further studies are certainly warranted, and a randomized controlled trial is currently in progress.

## Figures and Tables

**Table 1:** TLL fat harvest settings

Power	Temperature, °C	Pressure, psi	Target
Low	37	600	Soft fat, AFT, feathering
Medium	46	1100	Firm regular fat
High	55	1300	Fibrous fat, revision liposuction, etching

TLL indicates tissue liquefaction liposuction; psi, pounds per square inch; and AFT, autologous fat transfer.

**Table 2:** TLL procedures*

Harvest site	No. of procedures	Average lipoaspirate volume (mL)
Flanks	156	454 (150-1900)
Abdomen	114	770 (50-1750)
Thighs		
Anterior	31	919 (100-2500)
Posterior	10	430 (150-1200)
Medial	56	471 (100-1100)
Lateral	1	500
Posterior hips	13	360 (75-800)
Buttocks	7	1029 (300-1900)
Saddle bags	54	514 (200-1600)
Knees	28	504 (50-1200)
Anterior leg	2	300 (300)
Posterior leg	3	567 (300-1100)
Chest wall	34	367 (75-1650)
Bra line	20	356 (75-600)
Arms	19	550 (100-1200)
Neck	3	30 (20-50)
Submental	11	43 (15-75)

*The values given are number (range). TLL indicates tissue liquefaction lipoplasty.

**Table 3:** Complications experienced after TLL liposuction or AFT

Complication	n (%)
Harvest site (n = 255)	
Seroma	2 (0.78)
Hematoma	0
Donor site (n = 103)	
Fat necrosis	5 (4.85)
Oil cyst	1 (0.97)
Overall (n = 255)	
Cellulitis	2 (0.78)
Pain	0
DVT	0
Reoperation	1 (0.39)
Hospital admission	0

TLL indicates tissue liquefaction liposuction; AFT, autologous fat transfer; and DVT, deep venous thrombosis.
